# regionReport: Interactive reports for region-level and feature-level genomic analyses

**DOI:** 10.12688/f1000research.6379.2

**Published:** 2016-06-29

**Authors:** Leonardo Collado-Torres, Andrew E. Jaffe, Jeffrey T. Leek

**Affiliations:** 1 Department of Biostatistics, Johns Hopkins Bloomberg School of Public Health, Baltimore, Maryland, 21205, USA; 2 Lieber Institute for Brain Development, Johns Hopkins Medical Campus, Baltimore, Maryland, 21205, USA; 3 Center for Computational Biology, Johns Hopkins University, Baltimore, Maryland, 21205, USA; 4 Department of Mental Health, Johns Hopkins Bloomberg School of Public Health, Baltimore, Maryland, 21205, USA

**Keywords:** Report, Interactive, Reproducibility, Genomics, Sequencing, ChIP-seq, RNA-seq, Software

## Abstract

regionReport is an R package for generating detailed interactive reports from region-level genomic analyses as well as feature-level RNA-seq. The report includes quality-control checks, an overview of the results, an interactive table of the genomic regions or features of interest and reproducibility information. regionReport provides specialised reports for exploring DESeq2, edgeR, or derfinder differential expression analyses results. regionReport is also flexible and can easily be expanded with report templates for other analysis pipelines.

## Introduction

Many analyses of genomic data result in regions along the genome that associate with a covariate of interest. These genomic regions can result from identifying differentially bound peaks from ChIP-seq data ^1^, identifying differentially methylated regions (DMRs) from DNA methylation data ^2^, or performing base-resolution differential expression analyses using RNA sequencing data ^3, 4^, among other analysis pipelines. The genomic regions themselves are commonly stored in a GRanges object from GenomicRanges ^5^ when working with R or the BED file format on the UCSC Genome Browser ^6^. Other information on these regions, for example summary statistics on the magnitude of effects and statistical significance, also provide useful information and can be stored as metadata in GRanges objects. The usage of R in genomics is increasingly common due to the usefulness and popularity of the Bioconductor project ^7^, and in the latest version (3.3), 300 unique packages use GenomicRanges for many workflows, demonstrating the widespread utility of identifying and summarizing characteristics of genomic regions.

Bioconductor is particularly strong for differential expression analyses, with 206 packages using the Differential Expression BiocView. RNA-seq data is commonly used to perform feature-level analyses at either the transcript, gene or exon levels with Bioconductor packages DESeq2 ^8^ and edgeR ^9– 11^, among others. The features can also be expressed regions identified in an annotation-agnostic procedure by derfinder ^3^. In an exploratory data analysis of DESeq2 or edgeR results it is common to create a set of plots in order to identify potentially problematic samples or features. For example, in such an exploratory analysis it is common to use a dimension reduction technique such as principal component analysis to determine if samples are clustering by group or another variable of interest. This type of plot is useful for detecting artifacts, such as mislabeling of samples.

Here we introduce regionReport which allows users to explore genomic regions of interest, derfinder, DESeq2, and edgeR results through interactive stand-alone HTML reports that can be shared with collaborators. These reports are flexible enough to display plots and quality control checks within a given experiment, but can easily be expanded to include custom visualizations or text describing the main conclusions of the exploratory analysis. The resulting HTML report emphasizes reproducibility of analyses ^12^ by including all the R code without obstructing the resulting plots and tables. Alternatively, static PDF reports can be generated and easily shared among collaborators. We envision regionReport will provide a useful tool for exploring and sharing genomic region-based, DESeq2, and edgeR results from high throughput genomics experiments.

## Methods

### Implementation

**Figure 1.**
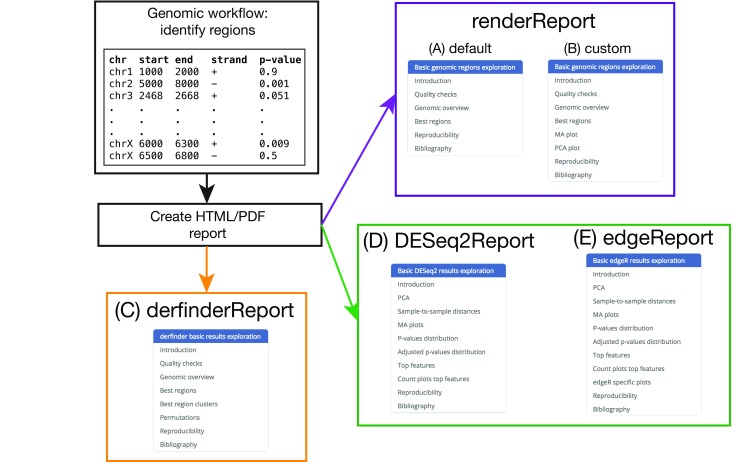
regionReport overview. Example region input, the appropriate regionReport function to use, and menu of the resulting report for: (A) the general use case, (B) a customised report, (C) derfinder results, (D) DESeq2 results and (E) edgeR results.

**Figure 2.**
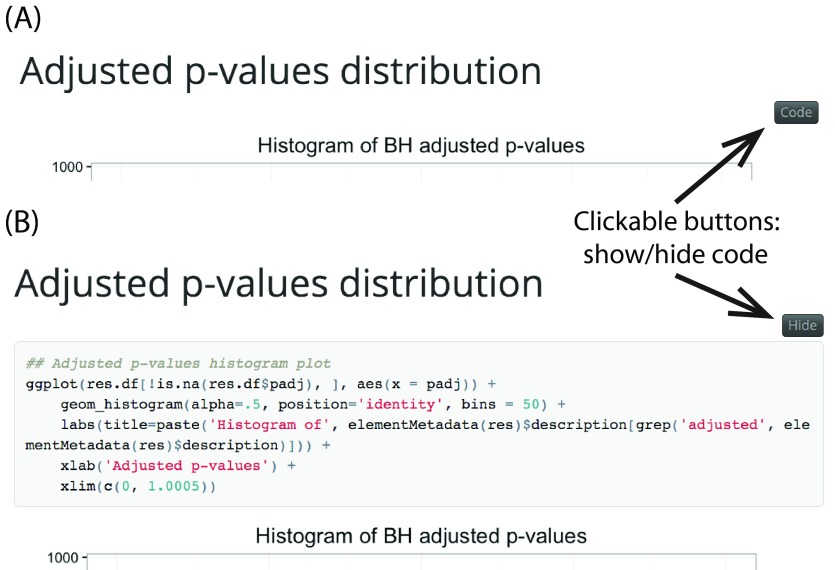
Interactively display the code for each table/figure in the report. (AB) View by default and (B) after clicking on the “code” toggle for a section in the report and the HTML reports include a toggle to hide/show all the R code.

The package includes R Markdown templates which are processed using rmarkdown ^13^ and knitr ^14^ to produce HTML or PDF reports. HTML reports can be styled using knitrBootstrap ^15^ or with rmarkdown templates that include interactive features. The regionReport package generates a report that includes a series of plots for checking the quality of the results and an interactive table with the best regions or features. Each element of the report has a brief explanation, although actual interpretation of the results is dataset- and workflow-dependent. To facilitate navigation a menu is included, which is useful for users interested in a particular section of the report. Figure 1A shows the menu of the general report for a set of regions with associated p-values. The code for each plot or table is hidden by default and can be shown by clicking on the “code” button as shown in Figure 2. Further customization of the reports can be done by providing custom code, changing the default plots, or by modifying the R Markdown templates included in regionReport.

## General region report

### Quality checks

This section of the report includes a variety of quality control steps which help the user determine whether the results are sensible. The quality control steps explore:

- P-values, Q-values, and FWER adjusted p-values
- Region width
- Region area: sum of single-base level statistics (if available)
- Mean coverage or other score variables (if available)

**Figure 3.**
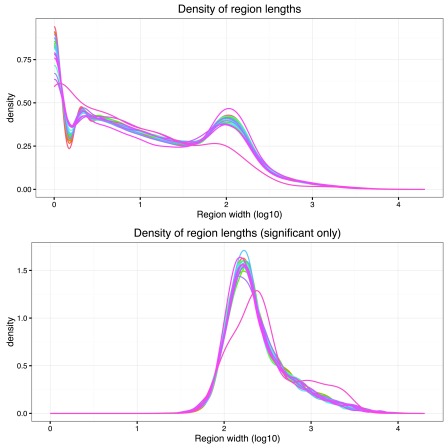
Distribution of region widths for all regions in the derfinder use case example with the *BrainSpan* dataset. The top figure shows the region width distribution for all regions while the bottom one shows it only for the significant regions. One line is shown per chromosome in each of the plots.

A combination of density plots and numerical summaries are used in these quality checks. If there are statistically significant regions, the distributions are compared between all regions and the significant ones. For example, the distribution region widths might have a high density of small values for the global results, but shifted towards higher values for the subset of significant regions as shown in Figure 3.

### Genomic overview

**Figure 4.**
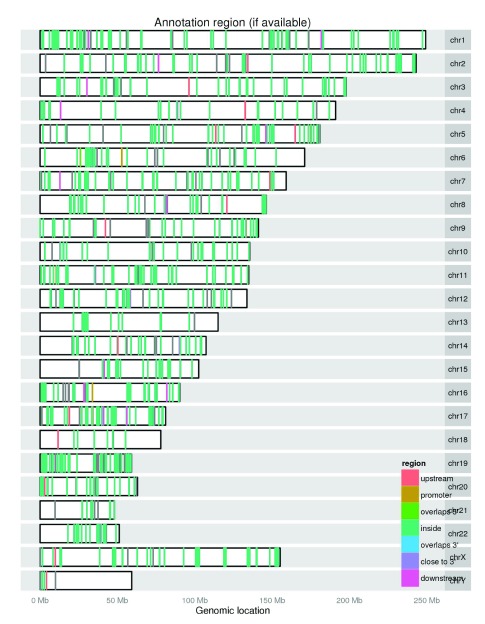
Genomic overview of the annotation type for the significant regions in the derfinder use case example with the *Hippo* dataset.

The report includes plots to visualize the location of all the regions as well as the significant ones. Differences between them can reveal location biases. The nearest known annotation feature for each region is summarized and visually inspected in the report. This type of plot can be useful to quickly check whether significant regions are concentrated in a chromosome or in an annotation type. For example, Figure 4 shows the annotation information for the significant regions with most regions contained inside genes, which is expected with RNA-seq data.

### Best regions

**Figure 5.**
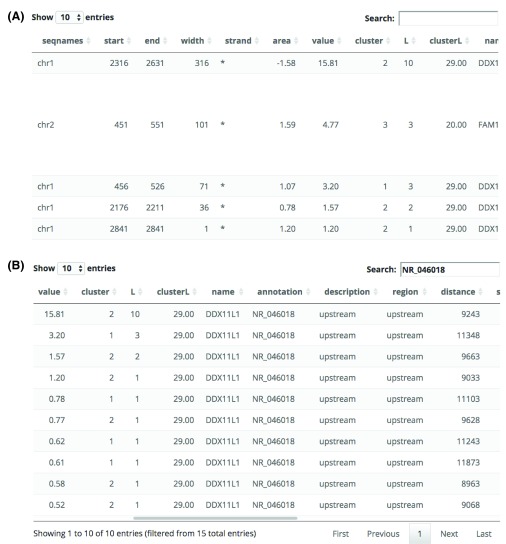
Interactive table with results for the top regions in the general use case example using bumphunter results. The interactive table can ( **A**) show all the top regions of ( **B**) a subset of the results by using the search box. The table can also be sorted by each of the different columns.

An interactive table with the top regions (500 by default) is included in this section as shown in Figure 5A. This allows the user to sort the region information according to their preferred ranking option. For example, lowest p-value, longest width, chromosome, nearest annotation feature, etc. The table also allows the user to search and subset it interactively as shown in Figure 5B. A common use case is when the user wants to check if any of the regions are near a known gene of their interest.

### Reproducibility

**Figure 6.**
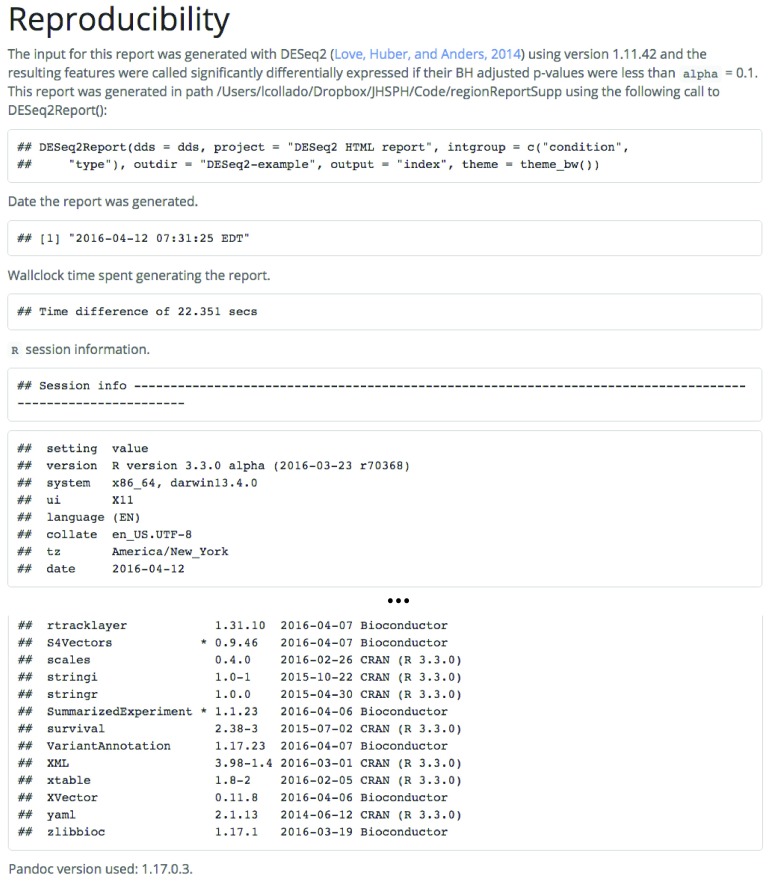
Reproducibility section for a report using DESeq2 results. The reproducibility information includes the actual function call used to generate the report, the path where the report was generated, the time it took to create the report, details about the R session information, and the pandoc version used for rendering the HTML report. For reports based on DESeq2 results, the version used to perform the differential expression analysis and cutoff used are also displayed. Note that DESeq2 version used for the analysis and for the report might differ.

At the end of the report, detailed information is provided on how the analysis was performed. This includes the actual function call to generate the report, the path where the report was generated, time spent, and the detailed R session information including package versions of all the dependencies. An example is shown in Figure 6 with the R package information truncated.

The R code for generating the plots and tables in the report is included in the report itself, thus allowing users to manually reproduce any section of the report, customize them, or simply change the graphical parameters to their liking.

### Customization

regionReport allows users to customize the reports to their liking. This can be done in different ways depending on the amount of customization the user is looking for. Several plots are made with ggplot2 and the user might want to change the default theme, for example to a black and white theme as shown in the function call in Figure 6. Another user might be interested in adding code that creates more plots than the ones included by default in the report. For example, the user might be interested in adding a MA and a PCA plot to the default report. This can be done via the customCode argument which results in new sections added to the menu as shown in Figure 1B compared to Figure 1A. Further customization can be achieved by modifying the templates included in regionReport and using the template argument.

### derfinder report

**Figure 7.**
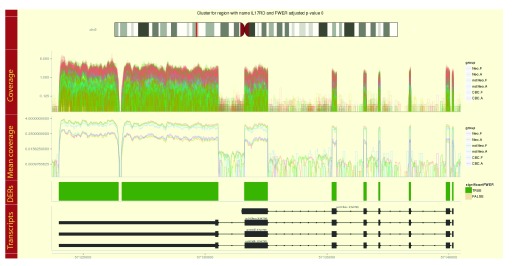
Example region cluster plot for the derfinder use case example with the *BrainSpan* dataset. Coverage curves are shown for each sample colored by their group membership. Mean coverage curves by group, differentially expressed regions (DERs) and known transcripts are shown in the remaining tracks.

When exploring derfinder results from the single base-level approach, for each of the best 100 (default) DERs a plot showing the coverage per sample is included in the report. These plots allow the user to visualize the differences identified by derfinder along known exons, introns and isoforms. The plots are created using derfinderPlot ^16^. Due to the intrinsic variability in RNA-seq coverage data or mapping artifacts, in situations where there are two candidate DERs that are relatively close there might be reasons to consider them a single candidate DER and its important to visualize them. This tailored report groups candidate DERs into clusters based on a distance cutoff. After ranking them by their area, for the top 20 (default) clusters it plots tracks with the coverage by sample, the mean coverage by group, the identified candidate DERs colored by whether they are statistically significant, and known alternative transcripts as shown in Figure 7. Figure 1C shows the main categories of the report generated from a richer region data set than in the general case.

### DESeq2 and edgeR reports

**Figure 8.**
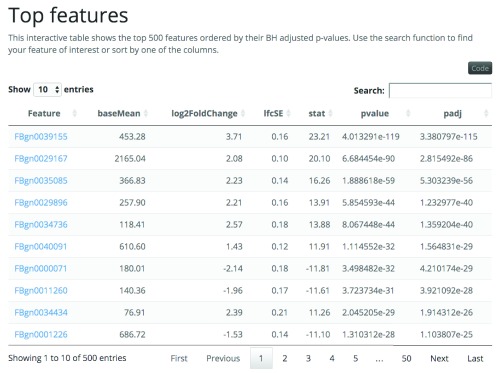
Interactive table for top features from the DESeq2 use case example.

Feature-level differential expression analyses result in a set of features (genes, exons) with a p-value for each feature. To perform such analyses, some phenotype information about the samples is usually available. With this information, you can explore the raw data to identify potentially problematic samples using principal component analysis and sample distance plots. You can also explore the results and check the features marked as differentially expressed with MA plots and a histogram of the p-values distribution. regionReport provides a template that allows you to create all these plots easily for DESeq2 results ( Figure 1D). It has similar components to the region-level reports such as an interactive table for the top features as shown in Figure 8, but also highlights specific exploratory plots for this type of results. regionReport can also be used for edgeR results ( Figure 1E) resulting in very similar reports given the internal implementation. The only difference is that reports for edgeR results include sections for visualizing the biological coefficient of variation and the multidimensional scaling plot of distances between feature expression profiles. See the use cases for example reports from DESeq2 and edgeR results.

### Operation

***Installation*.** regionReport and required dependencies can be easily installed from Bioconductor with the following commands:

source(“ http://bioconductor.org/biocLite.R”)

biocLite(“ regionReport”)

***Input*.** To generate the report, the user first has to identify the regions of interest according to their analysis workflow. For example, by performing bumphunting to identify DMRs with bumphunter. The report is then created using renderReport() which is the main function in this package as shown in Figure 1A,B.

For the derfinder use case, the derfinderReport() function creates the recommended report that includes visualizations of the coverage information for the best regions and clusters of regions. Similarly DESeq2Report() and edgeReport() create reports for DESeq2 and edgeR results, respectively.

***Output*.** A small example can be generated using:

example(“ renderReport”, “ regionReport”, ask=FALSE)

The resulting HTML file will open in the users default browser when using R in an interactive session. Note that alternative output formats such as PDF files can also be generated, although they are not as dynamic and interactive as the HTML format.

## Use cases

The supplementary website contains reports using DiffBind, bumphunter, derfinder, DESeq2, and edgeR results. The derfinder use case is illustrated with data sets previously described ^3^ with a moderately sized data set (25 samples), and a large data set with 484 samples. We encourage you to explore the following example reports:

- general HTML report example using bumphunter results,
- customized general HTML report using DiffBind results with histograms instead of density plots,
- DESeq2 HTML and PDF reports,
- edgeR HTML and PDF reports using the custom ggplot2 theme theme_linedraw(),
- edgeR-robust HTML report,
- HTML report using derfinder results with the *BrainSpan* dataset (484 samples) and styled with knitrBootstrap,
- HTML report using derfinder results with the *Hippo* dataset (25 samples) and styled with knitrBootstrap.

## Summary

regionReport creates interactive reports from a set of regions and can be used in a wide range of genomic analyses. Reports generated with regionReport can easily be extended to include further quality checks and interpretation of the results specific to the data set under study. These shareable documents are very powerful when exploring different parameter values of an analysis workflow or applying the same method to a wide variety of data sets. The reports allow users to visually check the quality of the results, explore the properties of the genomic regions under study, and inspect the best regions and interactively explore them.

Furthermore, regionReport promotes reproducibility of data exploration and analysis. Each report provides R code that can be used as the starting point for other analyses within a dataset. regionReport provides a flexible output for exploring and sharing results from high throughput genomics experiments.

## Software availability

### Software access

regionReport is freely available via Bioconductor at Bioconductor.org/packages/regionReport. The supplementary website http://leekgroup.github.io/regionReportSupp/ hosts the code and output for generating all the use cases described. Versions of all software used are included in the reports.

### Latest source code

The latest source code is available at github.com/leekgroup/regionReport. However, we highly recommend users to install regionReport directly from Bioconductor at bioconductor.org/packages/regionReport.

### Archived source code as at the time of publication

Archived source code available at dx.doi.org/10.5281/zenodo.55274

### License

Artistic-2.0.
