# Supplementary material for: regionReport: Interactive reports for region-level and feature-level genomic analyses
Source: F1000Res. 2016 Jun 29;4:105. Originally published 2015 May 1. [Version 2] doi: 10.12688/f1000research.6379.2 (PMC4934510; doi:10.12688/f1000research.6379.2)
Supplement: Peer review history

# Peer review: Referee response for version 2

**DOI:** 10.5256/f1000research.9717.r14687

**Published:** 2016-06-30

**Authors:** Timothy J. Triche Jr

**Affiliations:** 1 Jane Anne Nohl Division of Hematology, USC/Norris Comprehensive Cancer Center, Keck School of Medicine of the University of Southern California, Los Angeles, CA, USA

The authors have gone far beyond my expectations in addressing the lack of visual aids and examples in the original manuscript; the (new?) DiffBind example, as well as the excerpted figures from the BrainScan, clearly show strengths that derFinder and regionReport bring to bear when compared to existing tools.

 All of my previous reservations (and then some) have been thoroughly addressed and I believe the resulting manuscript is a strong argument for the authors' toolchain.

I have read this submission. I believe that I have an appropriate level of expertise to confirm that it is of an acceptable scientific standard.

# Peer review: Referee response for version 1

**DOI:** 10.5256/f1000research.6840.r8559

**Published:** 2015-06-22

**Authors:** David Robinson

**Affiliations:** 1 Lewis-Sigler Institute for Integrative Genomics, Princeton University, Princeton, NJ, USA

The authors present regionReport, an R package to produce interactive HTML reports from a genomic-region based analysis, such as those produced by derfinder, bumphunter or DiffBind. The report shows quality control summaries, interactive tables of the most significant regions, and information on how regions lie in exons, introns, and intergenic regions.  Both novice and expert genomicists are sure to gain much from this, and the paper clearly and concisely explains the software and its use, while providing useful examples and instructions.

The idea of producing common reports for a Bioconductor object is ingenious, and will hopefully inspire packages for other types of biological data. One of the great strengths of the package is the reproducibility practices it follows. For example, the section at the end of the produced report that shows reproducibility information, such as the original command, the session info, and the amount of time the report took to generate, is a great idea. (Indeed, the option to add sessionInfo() and timers could probably be baked into rmarkdown, or a thin wrapper thereof). Another strength is the use of modern knitr templates, such as expandable tables. Scientists who want to develop automated reports should use this package as a guide.

Overall my concerns are minor, and mostly concern the package rather than the paper, some of which I attempt to address in a GitHub pull request.

**In pull request**

- If the renderReport function leaves early (for example, if it is interrupted by the user hitting Stop) it strands the user's R session in a working directory. Using the on.exit function, as described here, lets R return to the original directory instead.
- The options for customization of the report are limited, by the customCode argument, to chunks between the main text and the reproducibility section. Genomicists may wish to take advantage of these reports while customizing some of their outputs. (For example, the authors of region-finding packages may wish to wrap renderReport with a customized template for their own objects). I've added a template argument in my pull request, and go over another suggestion below.

**Not in pull request**

- The `template` argument is a start towards greater customization, but a further improvement would be to allow the user to provide a list of customized internal chunks (for example, density-pvalue). As it is now, these are constructed in the renderReport function and cannot be altered without rewriting the entire function. This suggests finding a way to abstract them, such as bringing them in from a separate file, would be useful.
- As one example of an important customization I'd make: the reports show density plots of p-values and q-values, but in my experience genomicists are more accustomed to histograms (especially since bumps in density plots may be misleading, while histograms can get a better sense of which bumps are meaningful). I understand if the authors wish to keep it as a density plot, but if so I would appreciate a way to change it for my own use.

**Minor issues**

- The use of "smart quotes" in code within the PDF, such as source (“http://bioconductor.org/biocLite.R”), make it inconvenient to copy and paste them into an R terminal. If there's any way this could be remedied by the author or editors, it should.

I have read this submission. I believe that I have an appropriate level of expertise to confirm that it is of an acceptable scientific standard.

# Peer review: Referee response for version 1

**DOI:** 10.5256/f1000research.6840.r8558

**Published:** 2015-06-11

**Authors:** Karthik Ram

**Affiliations:** 1 Berkeley Institute for Data Science, University of California Berkeley, Berkeley, USA

Review of regionReport: Interactive reports for region based analysis.

This short software tool article describes a new R package, `regionReport`, available from Bioconductor that generates HTML reports which allows users to explore genomic regions and quickly scan quality control information. The reports also provide provenance of code used in the analysis, including detailed session information to facilitate as much reproducibility as possible.

The paper/report clearly describes functionality of the tool, potential use cases, and details on installation and operation. 

Suggestions for improvement

1. Given that you are describing an HTML application, it would be really helpful to include more screenshots/figures rather than just the one, and the workflow diagram. Most readers are unlikely to install the package immediately (see further comments on 3) and so it would help to make the value proposition clear. I would also suggest annotating these figures highlighting the key parts. Happy to approve the narrative itself after this revision.
2. Given that the package primarily generates html based reports, it would be of great value to have these files in the `gh-pages` branch of a GitHub repo, such that reports could be automatically made available under `https://USERNAME.github.io/repo/file.html` much like the page that describes the supplementary material. One way to easily enable this would be to use the functionality in the `git2r` package (disclosure: I am a coauthor on the package) to programmatically create a new branch (if it doesn't already exist), generate the report, then add those files and push to GitHub (assuming the same folder in under git revision control). Obviously I am not expecting the authors to add this suggestion to the current version of the package, but as something to consider for future versions.
3. Reduce the number of dependencies. `locfdr` is no longer on CRAN. On a slightly slower than normal connection (currently on travel) it took a fairly long time to track down and install all the dependencies. I'd recommend moving non-essential dependencies to suggests and using something like this to selectively install packages as needed using `requireNamespace(pkg, quietly = TRUE)`. It was disappointing to go down a rabbit hole of dependencies and still not be able to install and run examples. However, I found the report examples posted online (here: http://leekgroup.github.io/regionReportSupp/bumphunter-example/index.html and here: http://leekgroup.github.io/regionReportSupp/DiffBind-example/index.html) extremely useful. The use of Twitter bootstrap also adds a layer of a familiarity that I found extremely useful.
4. It would be nice to have the package generate a direct link to the bib file under the bibliography. 

I have read this submission. I believe that I have an appropriate level of expertise to confirm that it is of an acceptable scientific standard, however I have significant reservations, as outlined above.

# Peer review: Referee response for version 1

**DOI:** 10.5256/f1000research.6840.r8554

**Published:** 2015-05-18

**Authors:** Timothy J. Triche Jr

**Affiliations:** 1 Jane Anne Nohl Division of Hematology, USC/Norris Comprehensive Cancer Center, Keck School of Medicine of the University of Southern California, Los Angeles, CA, USA

Needs more figures to demonstrate why a user would choose this tool.  For example http://leekgroup.github.io/regionReportSupp/bumphunter-example/index.html (but even better would be to show an example, e.g. ITGB2 exon inclusion/exclusion or multiscale DMRs, where in our hands at least, nothing else short of IGV really does the job, and IGV doesn't do it that well.)  The software is a firm foundation but the writeup needs work if it is to be compelling and thus influence readers to try out an unfamiliar tools.

My apologies for being harsh, but without figures, an applied paper simply will not be read. I would be less harsh if the underlying work were not compelling enough to command broader interest.  A poor writeup will doom the work to obscurity.

I have read this submission. I believe that I have an appropriate level of expertise to state that I do not consider it to be of an acceptable scientific standard, for reasons outlined above.

# Response

**Published:** 2015-05-18

**Authors:** Jeffrey Leek

**Affiliations:** Johns Hopkins Bloomberg School of Public Health, USA

Thanks, we will update with more figures and expand the description. This is meant to be a short description of the software but we certainly appreciate the feedback on how to increase users. We will update the draft and respond shortly.
